# Do functional changes occur in the bladder due to bladder outlet obstruction? ‐ ICI‐RS 2018

**DOI:** 10.1002/nau.24076

**Published:** 2019-07-06

**Authors:** Ruud Bosch, Paul Abrams, Marcio Augusto Averbeck, Enrico Finazzi Agró, Andrew Gammie, Tom Marcelissen, Eskinder Solomon

**Affiliations:** ^1^ Department of Urology University Medical Centre Utrecht Utrecht The Netherlands; ^2^ Department of Urology Bristol Urological Institute Bristol United Kingdom; ^3^ Department of Urology Moinhos de Vento Hospital Porto Alegre Brazil; ^4^ Department of Experimental Medicine and Surgery, Department of Surgery, Policlinico Tor Vergata University of Rome “Tor Vergata” and Urology Unit Rome Italy; ^5^ Bristol Urological Institute Southmead Hospital Bristol United Kingdom; ^6^ Department of Urology Maastricht University Medical Centre Maastricht The Netherlands; ^7^ Department of Urology Guy's and St Thomas’ NHS Trust London United Kingdom

**Keywords:** bladder dysfunction, bladder outlet obstruction, detrusor contractility, overactive bladder, underactive bladder

## Abstract

Studies on bladder dysfunction (BD), more specifically functional‐urodynamic changes in the bladder as a result of bladder outlet obstruction (BOO) have been summarized for this TT. Based on available, but limited evidence from human studies a three‐stage model can be hypothesized to characterize BOO‐induced bladder remodeling: hypertrophy, compensation (increased detrusor contractility during the voiding phase, often in combination with filling phase detrusor overactivity) followed by the phase of decompensation [detrusor underactivity]. The time between the start of compensation and eventual decompensation seems to be determined by age of onset, severity, and type of obstruction and clinical mitigating factors such as vascular and metabolic problems. Understanding the relative contributions of these factors may allow the development of personalized timelines and probabilities for these obstructed patients.

## 1 INTRODUCTION

Most studies on functional‐urodynamic changes in the bladder as a result of bladder outlet obstruction (BOO) have been done in the aging male with benign prostatic obstruction (BPO). Other prevalent causes of BOO include bladder neck obstruction (BNO), posterior urethral valves (PUV), and urethral strictures (US). Conceptually, bladder dysfunction (BD) might be induced by BOO but alternatively could be pre‐existing; also, a combination of these factors might be possible. BPO‐ or BOO‐associated BD has been classified as follows: (a) detrusor overactivity (DO) with or without reduced bladder compliance, (b) detrusor underactivity (DU) or (c) mixed pattern.^1^ BD is clinically relevant, since it persists in up to one‐third of men after transurethral resection of the prostate.^2^

This leads to several questions as follows:

1. How might BOO lead to BD and more particularly what is its structural, histological and biochemical background?
2. Is BD reversible after treatment of BOO and if so, is this always the case? In other words, can the detrusor recover after treatment?
3. If detrusor function does not recover, why is this? Is there a certain period of exposure to BOO that predisposes to nonrecovery or decompensation of the bladder? And if so, how can this point of no return be characterized? Or is decompensation mainly related to acute or acute on chronic retention?

Is age of onset of BOO relevant? Are any clinical mitigating factors involved?

These are the proceedings of the Think Tank: “*Do functional changes occur in the bladder due to bladder outlet obstruction?*” from the 2018 International Consultation on Incontinence‐Research Society (ICI‐RS) meeting.

## 2 HOW MIGHT BOO LEAD TO BD AND MORE PARTICULARLY WHAT IS THE BIOCHEMICAL, HISTOLOGICAL, AND STRUCTURAL BACKGROUND TO BD?

Although in this Think Tank we focused on potential changes in humans, it is almost imperative to summarize biochemical and histological findings from animal research.

### 2.1 BOO and oxidative stress (evidence from animal studies)

In the detrusor, BOO leads to initial inflammatory response and ischemia which eventually results in progression to smooth muscle hypertrophy.^3^ The process of detrusor ischemia‐reperfusion which is associated with BOO leads to increased oxidative stress (OS) in the bladder wall.^4^, ^5^

Increased formation of reactive oxygen species and/or decreased antioxidant defense are associated with increased OS; this, in turn, may cause cell damage through apoptosis. Studies in rats have suggested the possibility to improve detrusor blood flow with the medical therapy,^4^ but it is still unclear whether these findings will translate to the human situation.

### 2.2 Upregulation of HIF‐1a and VEGF

Galvin et al^6^ demonstrated a significant time‐dependent upregulation of hypoxia‐inducible factor (HIF)‐1α and vascular endothelial growth factor (VEGF) in human bladder SMCs exposed to hypoxia. HIF‐1α was expressed mainly in stromal cells between muscle bundles and in the connective tissue beneath the mucosal layer, whereas urothelium and detrusor muscle showed no immunoreactivity. Interestingly, the HIF‐1α response was limited in a time‐dependent manner. Furthermore, the probability of HIF‐1α immunoreactivity was four times greater in men with BOO for <10 years, than in those with BOO for >10 years with an odds ratio of 4.25, suggesting that the bladder can compensate for the first few years but that the adaptive response declines thereafter. Moreover, the risk of identifying a high expression of HIF‐1α was four times higher in men with urinary retention.^7^ Barbosa et al^8^ compared VEGF gene expression in bladder specimens from Schäfer grade IV or patients with higher BPO, with age‐matched controls: patients with severe BPO showed a statistically significant VEGF overexpression.

### 2.3 Increased collagen content

Animal models support the hypothesis that bladder smooth muscle (detrusor) does not adapt to chronic BOO in the same way skeletal muscle tissue adapts to overloads. In the latter, there is organized and functional hypertrophy. In the detrusor, hypertrophy leads to changes in the expression of the myosin heavy chain isoform^9^, ^10^ and to significant changes in the expression of a variety of muscle proteins,^11^ generating a distinct phenotype in response to obstruction. A consequence of this change is an increase in collagen production, which may play a role in decompensation of bladder function.^12^, ^13^ Several studies have demonstrated the replacement of smooth muscle by collagen in humans; however, cause and effect relationships have not been established.^13^, ^14^, ^15^ Mirone et al^15^ carried out a case‐control study to assess histological characteristics of the bladder wall of 36 men with BPO and 28 patients with nonmuscle invasive bladder cancer (NMIBC; control group without lower urinary tract symptoms (LUTS) and with comparable age distribution). Biopsies of the bladder wall were done during transurethral resections. Interestingly, collagen deposition was higher in patients with BOO than in those with NMIBC (48% vs 17%, respectively; *P* < .001).^15^

It is known that bladder wall elasticity changes with the relative content of connective tissue, collagen, and muscle.^16^, ^17^ Therefore elastography might be useful to assess the collagen content of the bladder wall noninvasively and thus might serve as a proxy for detrusor muscle contractility.

### 2.4 Bladder or detrusor wall thickness

Thickening of the bladder wall as a result of BOO has been attributed mainly to smooth muscle cell hypertrophy and collagen deposition. Ultrasound noninvasively estimates bladder wall thickness (BWT), and its correlation with BOO. Some investigators have proposed detrusor wall thickness (DWT) as a more reliable parameter, although data from BWT and DWT have shown consistent results with good accuracy of both techniques in predicting BOO.^18^ Several studies showed a significant difference in BWT between obstructed and nonobstructed patients.^19^, ^20^, ^21^ Degree of obstruction, prostate volume, and IPSS seem to be positively correlated to BWT.

## 3 HOW DO HISTOLOGICAL AND BIOCHEMICAL CHANGES IN THE BLADDER CORRELATE WITH (CHANGING) URODYNAMIC FINDINGS?

Increased bladder pressure is the only urodynamic parameter that can be improved clinically. Although multiple treatment options exist for LUTS/BPO, mainly surgery, and to some extent α‐1 adrenergic antagonists and 5‐α reductase inhibitors, have been reported to improve BOO.^22^, ^23^ The model of structural bladder remodeling with longstanding BOO explains failures of surgical and medical therapies when applied too late in its natural history. Currently, only a few clinical markers of structural bladder remodeling are available, including increased estimated bladder or detrusor weight and endoscopic evidence of trabeculation. Consequently, molecular mechanisms of bladder remodeling in BOO remain unclear. Further issues deserving investigations are timing and reversibility of BOO‐induced bladder remodeling.

Studies on human tissues indicate that BOO induces molecular and morphological‐structural alterations in multiple bladder compartments, namely urothelium, suburothelium, detrusor SMCs, detrusor ECM, and neurons. Cyclic stretch increased pressure, and hypoxia has been shown to modulate multiple signaling pathways involved in these alterations. The initial inflammatory response and ischemia are followed by progression to smooth muscle hypertrophy.^3^ Combined with an increase of collagen content in the bladder wall^13^, ^15^ this results in increased bladder wall thickness (BWT).^20^ Apart from BOO, there is evidence that BWT or DWT is linked to urodynamic changes. DWT might, therefore, be a proxy for detrusor contractility. Rademakers et al^24^ reported that a combination of low DWT and large bladder capacity accurately defines the presence of detrusor underactivity. De Nunzio et al^25^ observed a correlation between increased DWT and detrusor overactivity. A summary of findings from these studies^24^, ^25^ that correlate detrusor or bladder wall thickness (DWT/BWT) with detrusor dysfunction is presented in Table 1A and 1B.

**Table 1:** Summary of findings from studies that correlate detrusor or bladder wall thickness (DWT/BWT) with detrusor dysfunction

A. Details of findings from a comparative cohort study by Rademakers et al^24^ in 143 treatment naive men aged ≥ 40 y (median 62 y), suffering from nonneurogenic LUTS, with or without detrusor underactivity (DU). This study correlates urodynamic parameters of DU with DWT.			
Definition of detrusor underactivity (DU)	Ultrasound determined measurement of DWT in DU vs non‐DU group	Noninvasive urodynamic parameters in DU vs non‐DU group	
		Max bladder capacity (voided volume + postvoid residual)	Postvoid residual
PVR > 30 mL AND no BOO OR dysfunctional voiding in the pressure‐flow study (33/143). These men had Wmax 4.4 (range 3.7‐6.0) W/m^2^	1.3 mm vs 1.9 mm (*P* < .001)	560 mL vs 385 mL (*P* < .001)	130 mL vs 71 mL (*P* = .027)
B. Details of the findings from a comparative cohort study by De Nunzio et al^25^ in 195 men, aged ≥45 y, suffering from LUTS without benign prostatic obstruction (Schäfer class <2 on the pressure‐flow study). This study correlates the urodynamic parameters of detrusor overactivity (DO) with BWT.			
Definition of detrusor overactivity (DO)		Ultrasound measurement of BWT in DO vs non‐DO men	
According to ICS report on standardization of terminology: 98 men with DO and 97 men without DO		4.3 ± 1.15 mm vs 3.6 ± 0.77 mm (*P* = .001)	

Abbreviations: BOO, bladder outlet obstruction; BWT, bladder wall thickness; DWT, detrusor wall thickness; PVR, postvoid residual urine.

A three‐stage model can be hypothesized to characterize BOO‐induced bladder remodeling in humans: hypertrophy, compensation (increased detrusor contractility during the voiding phase, often in combination with filling phase detrusor overactivity) followed by the phase of decompensation (detrusor underactivity).^26^, ^27^ Decompensation and progression to BD correlate with severity and duration of BOO.^28^, ^29^

## 4 WHAT IS THE ROLE OF CLINICAL MITIGATING FACTORS?

Averbeck et al^13^ demonstrated that diabetic patients had a significantly higher collagen content in bladder wall biopsies than nondiabetic men (*P* = .024). Diabetes mellitus type 2 was also associated with DU (*P* = .015). Furthermore, reduced compliance (*P* = .042) and increased postvoid residual urine (PVR ≥ 200 mL; *P* = .036) were associated with higher collagen to smooth muscle ratio. These outcomes provide further insights in the origin of diabetes‐related BD.^13^ Galvin et al^6^ demonstrated significant BOO related, time‐dependent upregulation of VEGF in human bladder SMCs exposed to hypoxia. Interestingly, upregulation of VEGF was particularly evident in subjects with risk factors for atherosclerosis.^6^

## 5 WHAT IS THE ROLE OF AGING AS A MITIGATING FACTOR?

In 1020 healthy male volunteers, De Zeeuw et al^30^ studied longitudinal changes in isovolumetric bladder pressure in response to age‐related prostate growth. Several 5‐year age cohorts were studied. For longitudinal analysis, three noninvasive urodynamic investigations (condom method) were performed during the 5‐year follow‐up period. Cross‐sectional analysis was based on the 35‐year age range of the subjects (38‐72 years, with flat age distribution). Prostate volume increased with 1.9% per year. Q_max_ decreased with 1.1% per year. P_max (condom)_ increased longitudinally, that is, during the 5‐year follow‐up, but not cross‐sectionally. This could imply that compensation is a relatively fast process taking place in approximately 5 years.^30^

## 6 WHAT IS THE ROLE OF AGE AT ONSET OF BOO AND WHAT IS THE ROLE OF (BOO) EXPOSURE TIME?

Although it is well established that BOO results in BD, it is not well established which factors influence the timeline to result in significant BD as well as the time to return to normal function following deobstruction. Cohorts of young boys with PUV demonstrate how variable both these timelines are. Due to prenatal scanning protocols, PUVs are typically diagnosed and treated in the first few months of life.

In perhaps the only study with prevalve ablation urodynamic studies during infancy, PUVs were associated with high detrusor overactivity (mean peak DO pressure of 60 cm H_2_O) in all of the 16 boys studied.^31^ Valve ablation took place between birth and age of 5 months. During follow‐up in the first 3 years of life, detrusor overactivity resolved, and bladder capacity increased by 65% of boys. This would suggest that these boys were treated during the compensation stage.

In another study, valve ablation was performed around the age of 3, and 12/15 boys had DO. However, some 4 years later, 7 had normal bladder function (capacity and pressures) but the others had persisting bladder dysfunction. Of the relatively late presenters, presenting around the age of 6, 6/9 were overactive at follow‐up but half of these resolved after a 3‐year follow up. So, the urodynamic pattern of obstruction‐induced detrusor overactivity during the filling phase, that is generally found soon after valve ablation, gradually changes to detrusor underactivity in many boys and this seems to be the rule after puberty. This series supports the hypothesis that long‐term filling phase detrusor overactivity in boys with PUV is associated with voiding phase detrusor underactivity/hypocontractility when the initial presentation is not within the first 6 months after birth. However, longitudinal 15‐year follow‐up from birth to puberty is needed to validate this concept.^32^ What we do not know in this group is what factors determine which trajectory the bladder function takes after valve ablation. How come that the bladders of these boys do not revert to normal but evolve into DU? Does this mean that these patients were treated too late, at the end of the compensation phase, where the point of no return was already reached? A summary of findings from these studies in infants, boys, and adolescents showing changes in urodynamic parameters of detrusor function (detrusor overactivity, bladder compliance, bladder capacity, and voiding detrusor pressure) with time,^31^, ^32^ is presented in Table 2A and 2B.

**Table 2:** Summary of findings from studies in infants, boys, and adolescents showing changes in urodynamic parameters of detrusor function (detrusor overactivity, bladder compliance, bladder capacity, and voiding detrusor pressure) with time

A. Details of findings from a cross‐sectional study by Holmdahl et al,^31^ in 16 infants and young boys aged 0‐5.5 (mean 1.3) months at presentation, showing evolution of urodynamic parameters of detrusor function after posterior urethral valve (PUV) ablation (with five requiring repeat valve ablation). Valve ablation was done 0.1‐6 (mean 1.9) months after presentation.					
Urodynamic findings					
Parameter	At presentation (n = 8)	Mean 4 mos (range 2.5‐6) after VAbl (n = 10)	Mean 12 mos (range 6.5‐14.8) after VAbl (n = 12)	Mean 20 mos (range 17.8‐22.2) after VAbl (n = 6)	Mean 36 (range 27.8‐44.9) mos after VAbl (n = 5)
Max amplitude of detrusor overactivity pressure (cm H_2_O)	60	58	57	39	37
With decreased detrusor compliance, %	50	30	8	0	0
Mean cystometric capacity (mL [range])	40 (24‐75)	–	–	–	231 (182‐375)
Max voiding pressure; mean (cm H_2_O [range])	162 (83‐230)	–	–	76 (34‐172)	
With residual urine >1/3 of capacit, %	75	–	–	–	50

B. Details of findings from a cross‐sectional study by De Gennaro et al^32^ in 30 young boys and (pre)adolescents, showing the evolution of urodynamic parameters of detrusor function after posterior urethral valve (PUV) ablation performed at a mean age of 5.6 mo in 27 and after the first year of life in 3. Urodynamic studies (UDS) before valve ablation were not performed.			
Urodynamic findings			
Parameters	Group A	Group B	Group C
	(n = 15)	(n = 10)	(n = 5)
	Mean age at 1st and last evaluation: 2.8 and 7.7 y	Mean age at 1st and last evaluation: 6.2 and 8.8 y	Mean age at 1st and last evaluation: 9.4 and 15.2 y
Change in number with normal findings at UDS	From 3/15 to 7/15	From 3/10 to 3/10	From 3/5 to 0/5
Change in number with detrusor overactivity	From ≤ 12/15% to 0%	From ≤ 6/10% to 0%	From ≤ 1/5% to 0%
Change in number with low bladder compliance:	From 0/15 to 1/15	From 1/10 to 0/10	From 1/5 to 0/5
Change in number with hypocontractility (defined as voiding pressure <50 cm H_2_O)	From 0/15 to 4/15	From 0/10 to 6/10	From 0/5 to 5/5
Change in mean max voiding pressure (cm H_2_O)	From 102.7 to 56.4	From 98.5 to 52.4	From 72.6 to 33

Another group was predicting the timeline to bladder dysfunction secondary to BOO would be very useful are the relatively young men with BNO. BNO is the cause in 40% to 50% of men under the age of 50 years, with obstruction. Predicting the timeline of potential BD might allow for the safe deferral of BNI, which is relevant for the preservation of fertility. In four studies, reporting on a combined total of 362 men, the mean symptomatic duration before clinical analysis was approximately 4 years. In 30% of 110 men, DO was found. This gives some insight into the possible timeline of the compensatory phase. However, since obstruction is often nonspecific in its symptomatic presentation it is uncertain how much we can rely on this possible timeline; it seems however that it took less than 4 years in about one‐third of these men to develop obstruction‐induced compensatory increased contractility.^33^, ^34^, ^35^, ^36^

**Figure 1:**
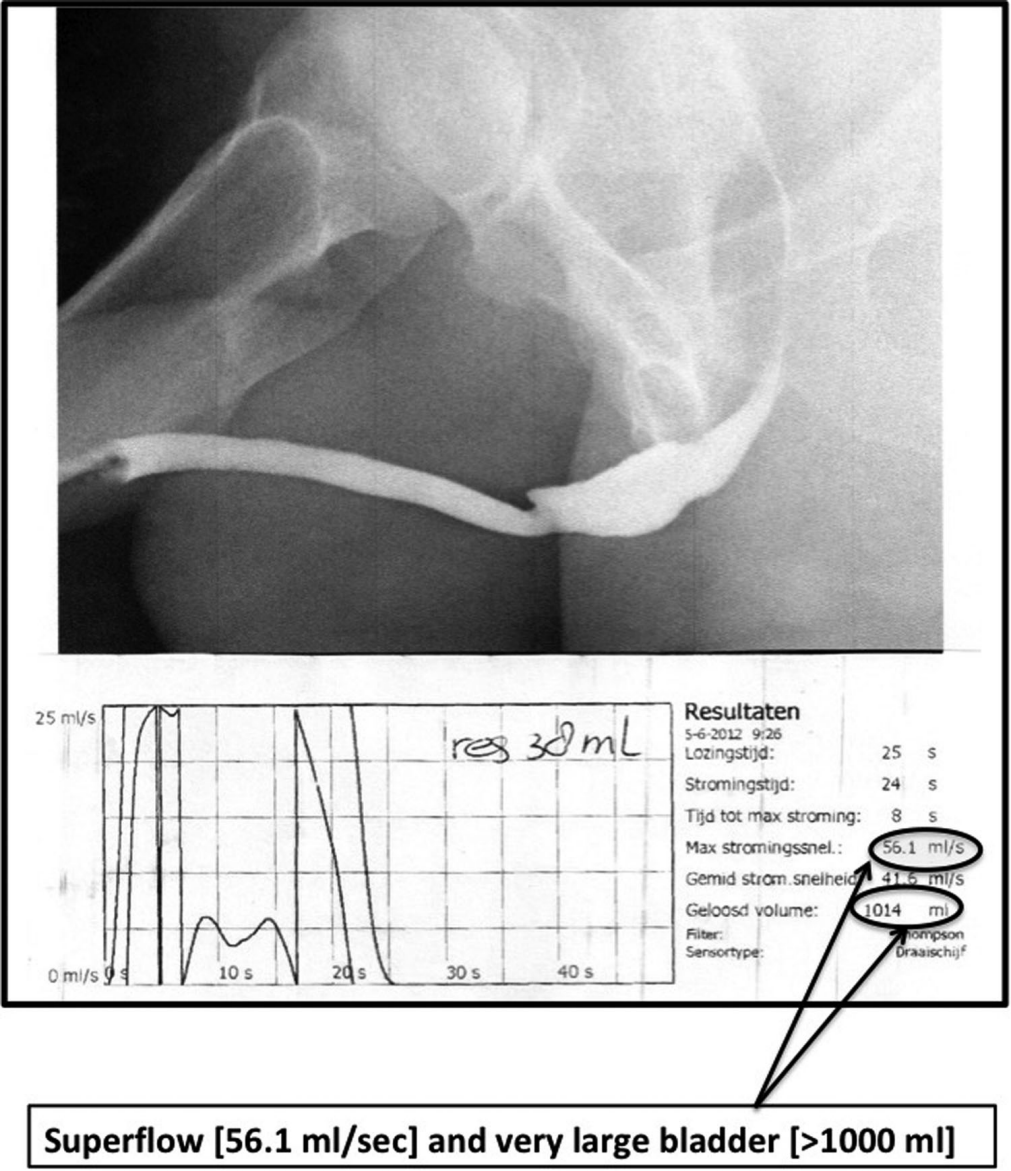
Voiding function after urethroplasty for bulbar stricture (dorsal buccal mucosa graft of the bulbar urethra). In this patient, there was a long period between the onset of stricture disease and the urethroplasty. He developed a very large bladder capacity. Six weeks after surgery the patient exhibits a super flow that subsequently normalized within 6 months

Some observations in relatively young men before and after surgery for urethral stricture disease are noteworthy. Before urethroplasty, some of these men have a very large bladder capacity (>900‐1000 mL) (Figure 1). Is this a sign of decompensation or is the detrusor wall just stretched, but not overstretched, due to voiding habits adapted to the BOO? Based on the findings of Rademakers et al^24^ it would be interesting to determine BWT/DWT in these men with large capacity bladders, particularly when they exhibit a superflow immediately after a successful urethroplasty. One would expect to see a relatively high DWT in these men. The superflow that is seen in a significant proportion of these men usually “normalizes” within a few months. Is the posturethroplasty superflow in the face of a suddenly disappearing obstruction, a sign of “compensation” by increased detrusor contractility? If so, then the normalizing flow could be a sign of “compensation” being reversed to normal. In case of a subject does not exhibit a superflow does that mean that the patient was already in the decompensation phase? Again, it would be interesting to determine BWT/DWT in these men and follow them while the flow rate changes from a superflow to normal flow, or assess the contractility using the isovolumetric pressure during a stop‐test.

From the data presented herein, it seems that the bladder starts to compensate for increased outflow resistance as soon as the bladder is exposed to the obstruction. The compensation phase can last for several years but thereafter the adaptive response declines and eventually the contractile function of the bladder declines and finally, the bladder decompensates. The available but limited evidence from human studies seems to indicate that the time between the start of compensation and eventual decompensation is determined by age of onset, severity, and type of obstruction and clinical mitigating factors such as vascular and metabolic problems. Understanding the relative contributions of these factors may allow the development of personalized timelines and probabilities for these obstructed patients.

## 7 WHEN AND UNDER WHICH CIRCUMSTANCES IS THE RECOVERY OF THE BLADDER POSSIBLE AFTER TREATMENT OF BOO?

Several studies have evaluated the effects of pharmacologic treatment for BPO on BWT. Treatment with α‐blockers has been shown to significantly reduce BWT in various studies.^37^, ^38^, ^39^ De Nunzio et al^40^ evaluated the effect of dutasteride add‐on therapy in patients with 27 “BPH” already being treated with α‐blockers.^40^ After 24 weeks of treatment, they observed a significant reduction in DWT. Unfortunately, no correlation with the results of pressure‐flow studies and DWT measurements have been reported.

Regarding BWT and surgery, one study evaluated 51 patients with “BPH” who underwent transurethral resection of the prostate: a very small but statistically significant reduction in DWT 1 month after surgery was found. Patients with larger prostates and a high degree of obstruction showed the greatest reduction.^41^

Functional changes in the bladder due to bladder outlet obstruction may not revert to normal after treatment: Thomas et al^42^ could not demonstrate any long‐term symptomatic or urodynamic gains from TURP in men with both BPO and detrusor underactivity. On the other hand, decompensation was rare in this relatively small and selected group of untreated BPO patients with DU.^42^ In the majority of older men presenting with large volume chronic retention and acute on chronic retention, bladder contractility may be beyond recovery as shown by a study that followed 228 men who were catheterized after going into acute retention: The cumulative rate of recurrent retention was 56% after 1 week and 68% after 1 year.^43^

## 8 SUMMARY

In a systematic review, Fusco et al^44^ have comprehensively summarized bladder outflow obstruction‐induced detrusor morphological alterations and related cellular events in humans, and the most important studies from that review have been highlighted by this Think Tank; however, correlation with urodynamic bladder contractility measures was not considered in that systematic review. To partially fill this gap we have summarized studies in humans correlating urodynamic detrusor dysfunction with detrusor/ bladder wall thickness (Table 1) and studies showing the evolution of urodynamic detrusor function after valve ablation in patients with posterior urethral valves (Table 2).

**Figure 2:**
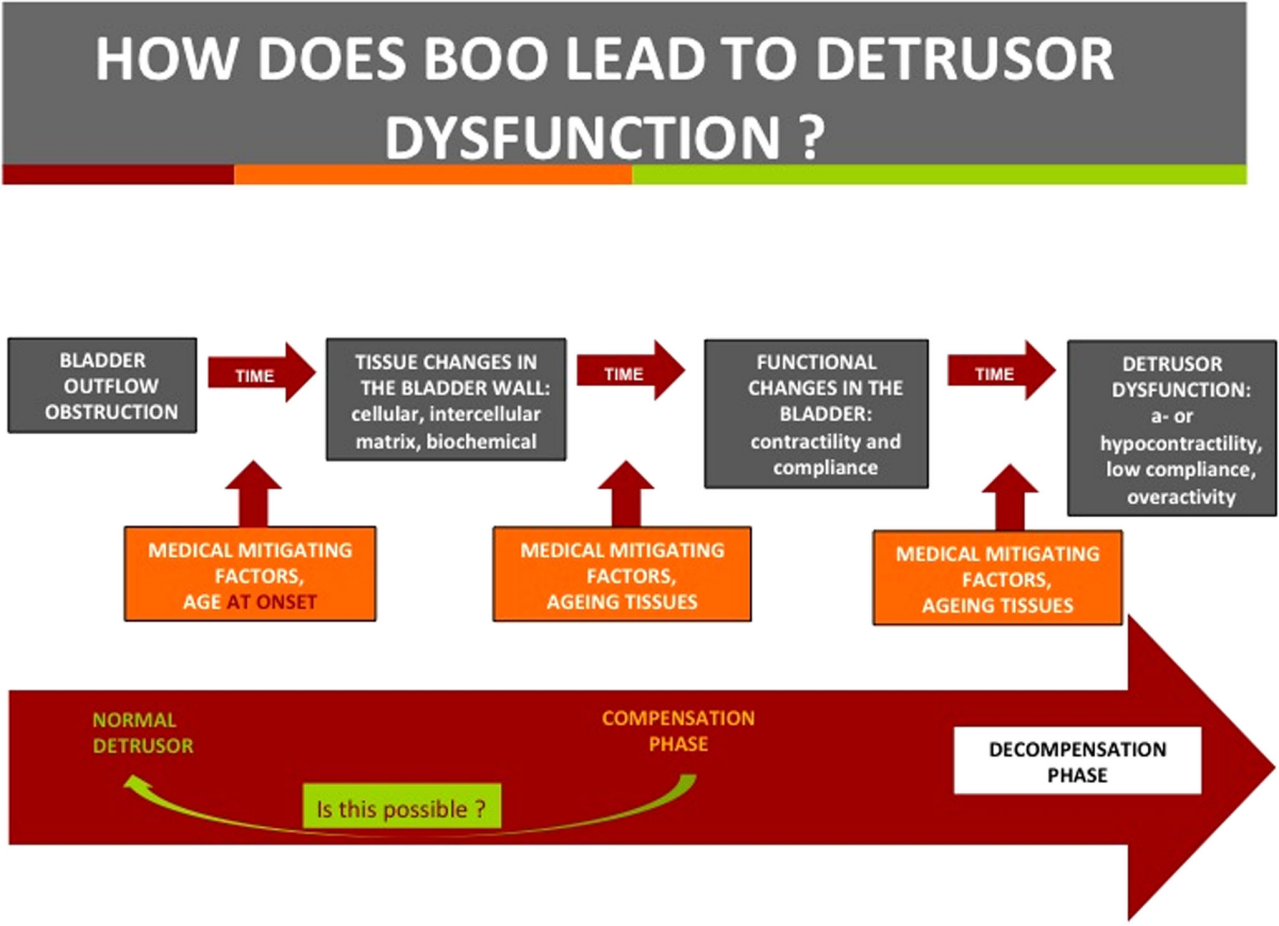
Phases involved in the development of detrusor dysfunction as a result of bladder outlet obstruction

Based on the review of the knowledge base related to the topics and questions that were addressed by this Think Tank, the possible phases involved in the development of detrusor dysfunction as a result of bladder outlet obstruction are summarized in Figure 2.

## 9 RESEARCH QUESTIONS

*How does BOO lead to detrusor dysfunction*? *Which human studies can be done* (*because animal studies either have limited relevance or are too expensive*)?

– Are there any noninvasive biomarkers for oxidative stress in humans?
– Could noninvasive techniques (eg, ultrasound assessment of bladder wall thickness, PVR, etc) identify which patients are at increased risk of BOO‐related bladder dysfunction? (*prospective studies are still needed)
– What is the role of urodynamics to define the management strategy (eg, medical vs surgical treatments for male LUTS) in high‐risk patients?

*How do histological and biochemical changes in the bladder function correlate with urodynamic findings*?

– What are the roles of HIFs as predictors in the early phase of changes?
– BWT measurements by US in cohorts such as men with urethral stricture disease before and after urethroplasty, with a correlation of the findings with duration and severity of urethral stricture disease.
– Can elastography measure collagen content of the bladder wall in vivo?.

*What is the role of clinical mitigating factors*? *What is the role of comorbidities* (*eg, diabetes mellitus, metabolic syndrome*) *in the development of BOO‐related bladder dysfunction*?

Effects of bariatric surgery on detrusor in terms of histology, biochemistry, and serial urodynamics.

*What is the role of* (*BOO*) *exposure time*? *How much time does it take before the bladder decompensates*?

This may be investigated by correlating bladder wall thickness and elasticity measurements with storage/voiding pressures in men with urethral stricture when BOO onset is known.

*What is the possible role of age at onset of BOO on the effect of exposure time*? *Does it take longer for a young bladder to decompensate*? *Are there groups of PUV patients with different outcome after valve ablation? Is there a tipping point between young and old age*?

Urodynamically compares cohorts of men that were older and younger at the onset of BOO.

*What is the role of aging as a mitigating factor?*

Study functional changes in the bladder in older women [without “confounder” of progressive obstruction].

*Is recovery possible after treatment of BOO? When and under which circumstances? In other words: if BOO is treated before decompensation, can the compensation phase be reversed?*

Cohorts after urethroplasty (with known stricture exposure time) that do and do not show initial superflow and subsequent normalization?

Role of PVR as a marker for decompensation.

## 10 CONCLUSIONS

Studies in humans and in animal models suggest a relationship between chronic BOO and both increased oxidative stress and increased collagen content in the bladder wall, which have been regarded as factors that trigger detrusor dysfunction.
